# Stimulatory Interactions between Human Coronary Smooth Muscle Cells and Dendritic Cells

**DOI:** 10.1371/journal.pone.0099652

**Published:** 2014-06-16

**Authors:** Sara Paccosi, Claudia Musilli, Roberto Caporale, Anna Maria Grazia Gelli, Daniele Guasti, Ann Maria Clemente, Maria Gabriella Torcia, Amelia Filippelli, Paolo Romagnoli, Astrid Parenti

**Affiliations:** 1 Department of Health Sciences, Clinical Pharmacology and Oncology Unit, University of Florence, Florence, Italy; 2 Central Laboratory, Azienda Ospedaliero-Universitaria Careggi, Florence, Italy; 3 Department of Experimental and Clinical Medicine, University of Florence, Florence, Italy; 4 Department of Experimental and Clinical Biomedical Sciences, University of Florence, Florence, Italy; 5 Department of Medicine and Surgery, University of Salerno, Salerno, Italy; University of Catanzaro Magna Graecia, Italy

## Abstract

Despite inflammatory and immune mechanisms participating to atherogenesis and dendritic cells (DCs) driving immune and non-immune tissue injury response, the interactions between DCs and vascular smooth muscle cells (VSMCs) possibly relevant to vascular pathology including atherogenesis are still unclear. To address this issue, immature DCs (iDCs) generated from CD14^+^ cells isolated from healthy donors were matured either with cytokines (mDCs), or co-cultured (ccDCs) with human coronary artery VSMCs (CASMCs) using transwell chambers. Co-culture induced DC immunophenotypical and functional maturation similar to cytokines, as demonstrated by flow cytometry and mixed lymphocyte reaction. In turn, factors from mDCs and ccDCs induced CASMC migration. MCP-1 and TNFα, secreted from DCs, and IL-6 and MCP-1, secreted from CASMCs, were primarily involved. mDCs adhesion to CASMCs was enhanced by CASMC pre-treatment with IFNγ and TNFα ICAM-1 and VCAM-1 were involved, since the expression of specific mRNAs for these molecules increased and adhesion was inhibited by neutralizing antibodies to the counter-receptors CD11c and CD18. Adhesion was also inhibited by CASMC pre-treatment with the HMG-CoA-reductase inhibitor atorvastatin and the PPARγ agonist rosiglitazone, which suggests a further mechanism for the anti-inflammatory action of these drugs. Adhesion of DCs to VSMCs was shown also *in vivo* in rat carotid 7 to 21 days after crush and incision injury. The findings indicate that DCs and VSMCs can interact with reciprocal stimulation, possibly leading to perpetuate inflammation and vascular wall remodelling, and that the interaction is enhanced by a cytokine-rich inflammatory environment and down-regulated by HMGCoA-reductase inhibitors and PPARγ agonists.

## Introduction

Inflammatory mechanisms play a key pathogenic role in arterial wall remodelling in response to different types of injury - including atherosclerosis, occasional or surgical trauma and arteritis [1]–[3] - and may lead to worsening and complication of disease [1], [4]. The geometry of the process, whether involving the whole circumference of the vessel or only part of it and where and how far along the vessel axis, depends primarily on the type of injury or dysregulation of the underlying mechanisms. The secretion of pro-inflammatory factors and direct intercellular interaction both cooperate to drive the process, but the regulatory pathways are not yet understood in detail.

A consequence of inflammation in the arterial wall is hypertrophy of intimal tissue, leading to wall thickening and eventually to stenosis of the lumen [3], [5]. The onset and progression of this process include functional changes in endothelial cells, T lymphocytes, monocyte-derived macrophages and vascular smooth muscle cells (VSMCs) [1], [2], [5]. Activation of all these cells leads to the generation of a wide spectrum of hydrolases, cytokines, chemokines, adhesion molecules and growth factors, together with lipid accumulation and proliferation of VSMCs and fibroblasts [1]. Immunohistochemical studies have revealed mononuclear cell infiltration and accumulation of activated T cells in early and late atherosclerotic lesions [6]. In these conditions, VSMCs are stimulated and show altered expression of transcription factors, growth factors, apoptosis-regulating genes, integrins, proteases and extracellular matrix proteins. They acquire increased capacity to proliferate, migrate, and secrete great amounts of extracellular matrix proteins [7].

Dendritic cells (DCs), the professional antigen presenting cells of the immune system, are candidate to a major role in the onset and progression of inflammation, even independent of specific immune responses. They are present in the normal human arterial intima and adventitia as part of vascular-associated lymphoid tissue (VALT), consisting of accumulations of immunocompetent and antigen presenting cells, which screen the microenvironment for potentially harmful antigens and drive inflammatory responses [8]. Many DCs localize to the neointima of atherosclerotic lesions together with T-lymphocytes, NK cells, mast cells and neointimal VSMCs [6], [8], [9]–[11]. Dendritic cells have not yet been found in normal arteries of laboratory rodents, but appear in response to atherogenic stimuli [12] and traumatic injury [2], [13], [14]. Endothelial dysfunction stimulates DC migration and adhesion [15] and the same occurs upon increased vascular oxidative stress [16].

The response of vascular smooth muscle cells to the inflammatory microenvironment of diseased arteries has been the object of several studies. Notwithstanding, the possible role of VSMCs in retaining and activating DCs in atherosclerotic lesions and, reciprocally, that of DCs in activating VSMCs to the altered functional state characteristic of those lesions are still unclear. This study was aimed at investigating the interaction of human coronary smooth muscle cells and human DCs *in vitro* and the possible influence of an inflammatory environment on this interaction. Since intercellular adhesion was a key result, we also addressed whether this finding could be replicated *in vivo*, in an experimental model of vascular wall inflammation in the rat.

## Methods

This study was approved by the Institutional Ethical Committee of the Azienda Ospedaliero-Universitaria Careggi, Firenze (prot. N. 2011/0034455 rif. N. 15/11); written informed consent was obtained by healthy donors.

### Coronary artery smooth muscle cell culture

Human coronary artery smooth muscle cells (CASMCs, lots 0000184180 and 0000169150) were purchased from Lonza (Walkersville, MD) which characterized them by positive immunostaining for alpha-smooth muscle actin (α-SMA) and negative immunostaining for factor VIII. Cells were grown in smooth muscle basal medium (SmBM) supplemented with hEGF, insulin, hFGF-B and gentamin/amphotericin-B (SmGM-2 SingleQuots; Lonza), 5% (v/v) heat inactivated fetal bovine serum (Hyclone Defined FBS; Thermo Ficher Scientific, Waltman, MA), 100 U/mL penicillin, 100 µg/mL streptomycin and 2 mmol/L glutamine (Sigma-Aldrich, St Louis, MO, USA), in a humidified atmosphere with 5% CO_2_ in air. Culture medium was changed every 2 days. Experiments were performed using cells between the 5^th^ and 10^th^ passage.

### Generation and maturation of DCs

Following a previously published method [17], mononuclear cells were isolated by Ficoll/Paque density gradient centrifugation from buffy coats obtained from healthy donors; written informed consent was obtained upon approval by the local Ethical Committee (prot. N. 2011/0034455 rif. N. 15/11). Micromagnetic-selected CD14^+^ cells were cultured in RPMI-1640 with 1000 U/mL rGM-CSF (Leucomax; Sandoz-Wander Pharma, Bern, CH) and 1000 U/mL IL-4 (R&D System, Minneapolis, MN, USA) for 6 days to generate immature DCs (iDCs). On day 7, non-adherent cells were harvested and analysed by flow cytometry technique (FACScanto; BD Biosciences, Bedford, MA, USA). To induce maturation, iDCs were stimulated with 10 ng/mL TNFα, 1000 U/mL IL-6 and 10 ng/mL IL1-β for 24 h and the resulting mature DCs (mDCs) were analysed by flow cytometry. The following fluorescent monoclonal antibodies were used for flow cytometry analysis of iDCs and mDCs: CD80-phycoerythtrin (PE), CD86-fluorescein isothiocyanate (FITC), CD86-allophycocyanin (APC; for blocking experiments), CD83-PE, DC-SIGN-PE, HLA-DR-FITC, CD38-PE, CCR-7-FITC (BD Pharmingen; San Diego, CA). In order to block Fc-mediated unspecific binding, cells were pre-incubated with serum and PBS (1∶1) for 30 min at 4°C. Isotype-matched antibodies were used as negative control. 7-amino-actinomycin D (7-AAD; Sigma-Aldrich, St Louis, Mo, USA) was used to exclude dead cells from analysis.

### Co-culture of CASMCs and iDCs

Immature DCs (iDCs) derived from CD14^+^ cells and CASMCs were co-cultured for 36 h in RPMI with 10% Hyclone Defined FBS (Thermo Fisher Scientific) without the maturation cocktail. In detail, in order to address the role of soluble factors released by the co-culture, CASMCs were plated in 6 multiwells, in which transwell chambers with 0.4 µm pore sized membranes were added (Millicell inserts; Merk-Millipore, Darmstadt, Germany). Immature DCs were plated into transwell chamber, in order to avoid any direct contact between the two types of cells while allowing medium sharing. After 36 h of co-culture in air with 5% CO_2_ at 37°C, DCs were collected and analysed by flow cytometry and mixed lymphocyte reaction (MLR). In some experiments CASMCs were treated for 24 h with inflammatory cytokines, *i.e.* 50 ng/mL TNFα (R&D System) and 50 ng/mL IFNγ (Peprotech, London, UK) before co-culturing with DCs. In the blocking experiments, co-cultures of iDCs and CASMCs were set up in the presence of neutralizing monoclonal antibodies against IL-6 (Merk-Millipore), TNFα and MCP-1 (R&D Systems). Mouse monoclonal IgG2b and IgG1 negative controls were from Merk-Millipore.

Co-cultures were also set up in serum-free medium, in order to exclude any confounding factors contained in the serum during DC maturation. Briefly, monocyte-derived immature DCs (iDCs) were co-cultured with CASMCs, inside transwell chambers, in RPMI with or without FBS. Obtained DCs were analysed by flow cytometry.

### Animal studies

The investigation conformed with the *Guide for the Care and Use of Laboratory Animals* (NIH Publication No. 85-23, revised 1996) and with the Italian (D.L. 116/92) and European Community (Directive 86/609/EEC, in OJ L 358 of December 18, 1986) guidelines on the use and protection of animals in experimental research. The study was approved by Ethical Committee For Animal Research of the Department of Pharmaceutical and Biomedical Sciences, University of Salerno, in Fisciano, SA, Italy on 8^th^ September 2011. Adult male Wistar rats (200–250 g) were obtained from Harlan Italy (San Pietro al Natisone, Udine, Italy). All rats were acclimated in individual cages for 1 week before experimentation and appeared healthy at visual inspection. They were maintained on a 12 h light/dark cycle at 24°C, at a relative humidity of 60%, and received food and water *ad libitum*. They were anaesthetized for surgery and for sacrifice by intraperitoneal injection of ketamine hydrochloride (80 mg/kg). Following a procedure described in detail elsewhere [18] the rats (three for each time point) were anesthetized and given an intravenous dose of ticarcillin (50 mg/kg). Through an incision in the anterior neck region a plastic Scanlan clamp for coronary artery bypass grafting was applied on a carotid for 10 s and a full thickness, 0.5 mm longitudinal incision was made through the carotid wall. Haemostasis was obtained with a single adventitial 8.0-gauge polypropylene stick. The other carotid was left uninjured, as control. Injured and uninjured carotids, 7, 14 and 21 days after injury, were analysed from three rats for each time point; three uninjured rats were used as further controls. Each animal represents an experimental unit.

### Isolation of human lymphocytes and mixed lymphocyte reaction (MLR)

Allogeneic peripheral blood mononuclear cells were seeded in RPMI 1640 and 10% FBS and monocytes were let to adhere. Leukocytes remained in suspension were collected, centrifuged at 1200 rpm for 10 min and used for MLR, as previously published [19]. Briefly, lymphocytes were seeded into 96-well plates at 10^5^ cells/well and purified allogeneic DCs (5×10^3^) were added to each well and incubated for 5 days at 37°C in a humidified chamber in air and 5% CO_2_. For the last 18 h, 1 µCi of [methyl-^3^H]thymidine (Amersham Pharmacia Biotech, Little Chalfont, UK) was added to each well. Cells were finally collected by a Filtermate Cell Harvester (Perkin–Elmer, Waltham, MA, USA). ^3^H-Thymidine uptake was quantified by liquid scintillation counting on a TopCount NXT Microplate Scintillation Counter (Perkin–Elmer). Each experiment was performed in triplicate.

### Electron microscopy

Cells in single culture were fixed upon suspension in medium. Co-cultures of DCs adhering to CASMCs were fixed, then removed with a scraper and pelletted. Rat carotids were fixed by immersion. All samples were fixed in 2% formaldehyde and 2.5% glutaraldehyde in 0.1 M cacodylate buffer, pH 7.4, osmicated and embedded in epoxy resin. Sections were stained with lead acetate and uranyl acetate and observed in a Jeol JEM 1010 electron microscope (Tokyo, Japan), at 80 kV, to assess the presence, localization and reciprocal contacts of VSMC and DCs.

### Immunohistochemistry

Cytospins of iDCs and mDCs were fixed with acetone for 5 min at room temperature. After blocking non-specific binding sites with 10 ng/mL bovine serum albumin (Sigma-Aldrich) in 0.1 M phosphate-buffer, pH 7.4, the slides were tagged with primary antibodies against CD207/langerin (IgG2b, clone 12D6; Novocastra Laboratories, Newcastle, UK), 1∶100, and DC-SIGN (polyclonal; Sigma-Aldrich), 1∶50, overnight at room temperature, followed by FITC-labelled, goat anti-mouse or anti-rabbit, polyclonal antibodies (Sigma-Aldrich), 1∶50, for 60 minutes at 37°C. Omission of primary antibodies or substitution with irrelevant ones were used as negative controls. Skin sections including epidermis (containing langerin positive, DC-SIGN negative Langerhans cells) and dermis (containing langerin negative, DC-SIGN positive connective tissue dendritic cells) were used as positive controls. The slides were mounted with Gel/Mount (Biomeda, Foster City, CA, USA), observed in an Axioskop microscope equipped for epifluorescence (Zeiss, Oberkochen, Germany) and photographed with an Axio Vision 4 system with a digital multichannel fluorescence module and dedicated software (Zeiss).

For α-SMA detection in CASMCs, cells grown on coverslips were fixed with 2% phosphate-buffered formaldehyde and labeled with anti–human α-SMA monoclonal antibody (1∶50, Dako A/S, Denmark), overnight at 4°C, followed by an Alexa Fluor 594 conjugated goat-anti mouse secondary antibody (1∶250, Molecular Probes, Oregon, USA), for 1 h at room temperature. CASMCs expressed α-SMA as expected from Lonza indications (not shown).

### Real- time PCR

Cells were seeded at 80% confluence onto 6 cm-diameter Petri dishes in SmBM medium supplemented with cytokines, growth-factors and 5% (v/v) FBS, starved for 24 h (using only medium and 0.1% FBS) and then stimulated for 24 h with TNFα or IFNγ (50 ng/mL either cytokine). Total RNA was extracted using TRI-reagent (Sigma-Aldrich) according to manufacturer's instructions and 1 µg of total RNA was reverse transcribed using Stratagene kit (Quiagen, Venlo, Netherlands) and random primers. Quantitative real-time PCR was performed by incubating sample cDNA or control cDNA in triplicate with Sybr Green PCR Master MIX (Superarray Bioscience, Frederick, MD, USA) and primers specific for genes to be tested and for *GAPDH* as housekeeping gene in 96 well-microtiter plates. *ICAM-1, VCAM-1* and *GAPDH*-specific primers (Table 1) were obtained by Life Technologies (Carlsbed, CA, USA). PCR was performed on an ABI PRISM 7900 (Applied Biosystems, Termo Fisher Scientific) as follows: 50°C for 2 min, 95°C for 10 min followed by 40 cycles of 95°C for 15 s and 60°C for 1 min. Results for target genes were normalized to *GAPDH* expression. Fold increase in mRNA was calculated with the 2^−ΔΔCT^ method.

**Table 1:** Primers and conditions for real time-PCR.

RefSeq	Description	Sequence (5′->3′)	Band size
NM_001078	hVCAM-1, forward	GGCAGGCTGTAAAAGAATTGCA	*bp 66*
	hVCAM-1, reverse	TCATGGTCACAGAGCCACCTT	
NM_000201.2	hICAM-1, forward	CAGACAGTGACCATCTACAGCTT	*bp 102*
	hICAM-1, reverse	TTCTGAGACCTCTGGCTTCGT	
NM_002046.3	hGAPDH, forward	Not provided by Applied Biosystems	*Bp 118*
	hGAPDH, reverse	Not provided by Applied Biosystems	

### Cell migration

A modified Boyden chamber (48-multiwell plates; Neuroprobe, Gaithersburg, MD, USA) was used for chemotaxis studies [20]. Polyvinyl-pyrrolidone-free polycarbonate filters, 8 µm pore size, were coated with 100 µg/mL collagen type I and 10 µg/mL fibronectin (BD Biosciences, Bedford, MA, USA). Conditioned medium of mDCs or ccDCs was prepared from 1×10^6^/ml mDCs or ccDCs/mL cultured in RPMI-1640 containing 10% FBS for 2 days. The supernatants were collected, cleared of cells and debris by centrifugation at 550 g for 5 min, aliquoted and stored at -20°C until use. Some experiments were performed with media recovered from mDCs and co-cultures grown in FBS-free medium, in order to assess the influence of serum on cell migration. Conditioned medium was added to the lower wells, while cells (12×10^3^) in 50 L medium with 1% FBS were seeded into the upper wells of the chamber, which was incubated at 37°C for 6 h. Methanol-fixed cells were stained with Diff-Quik (Dade Behring, Dudingen, CH) and cell migration was measured by microscopic evaluation of the number of cells moved across the filter, in ten randomly selected fields at magnification 400x. Each experimental point was measured in triplicate. In neutralizing experiments, CASMCs were let to migrate towards medium conditioned by ccDCs in the presence of the neutralizing monoclonal antibodies against IL-6, MCP-1 and TNFα𝛂 mentioned above for adhesion assay. For statistical analysis each well was assumed as a sample unit. The data were normalized for each experiment by expressing the number of migrating cells as per cent of the average value in the corresponding unstimulated wells. Since the results were distributed approximately symmetrically, they were subjected to statistical analysis without further transformation.

### Multiplex assay

Supernatants of iDCs, mDCs, ccDCs and CASMCs were recovered, centrifuged at 2500 rpm for 5 minutes and then analyzed using Milliplex Map Kit technology (Merk-Millipore) on BIOPLEX apparatus (Bio-Rad, Hercules, CA, USA) following manufacturer's recommendations, for the simultaneous quantification of TNFα, IL-6, IL-1β, INFγ, MCP-1. For mDCs and ccDCs the supernatant was analysed upon 36 h culture or co-culture respectively, *i.e.* upon DC maturation.

### Adhesion assay

The adhesion of mature (mDCs) to CASMCs was analysed by fluorescent assay using Vybrant Cell Adhesion Assay Kit (Invitrogen, Carlsbad, CA, USA) [21]. Briefly, DCs were pulsed with 5 µmol/L calcein and incubated 45 min with a monolayer of CASMCs, which had been pre-treated for 12, 24 or 36 h with TNFα or IFNγ, either one 50ng/mL, in SmBM with 1% (v/v) FBS but without growth factors. After washing, adherent cells were counted with a Victor 3 fluorescence multireader (Perkin Elmer) [21]. To assess the effect of neutralizing antibodies, DCs were pre-treated with anti-CD18 (0.5 µg/well, BioLegend, San Diego, CA, USA), anti-CD11c (0.5 µg/well, Millipore, Milan, Italy), or anti-DC-SIGN (0.5 µg/well, R&D Systems) neutralizing monoclonal antibodies or with non immune IgG2b and IgG1 (Merk-Millipore), for 1 h. To assess the effect of atorvastatin and rosiglitazone on cell adhesion, CASMCs were pretreated with test drugs and then stimulated with TNFα and IFNγ for 24 h. After 24 h, CASMCs were washed twice and co-incubated for 45 min with mDCs to assess cell adhesion.

### Statistics

Data are reported as mean ± standard error (SE). Differences between two groups were determined by Student's t test for unpaired data. Differences between multiple groups were determined by ANOVA followed by Tukey's multiple comparison test. P values <0.05 were recorded and considered as significant. In experiments on cell migration, the correlation between variables was evaluated by Spearman's *r* test. The program Prism 5.0 (GraphPad Software, La Jolla, CA, USA) was used for all statistical analyses.

## Results

### Coronary artery SMCs stimulate the maturation of iDCs phenotype and function

**Figure 1:**
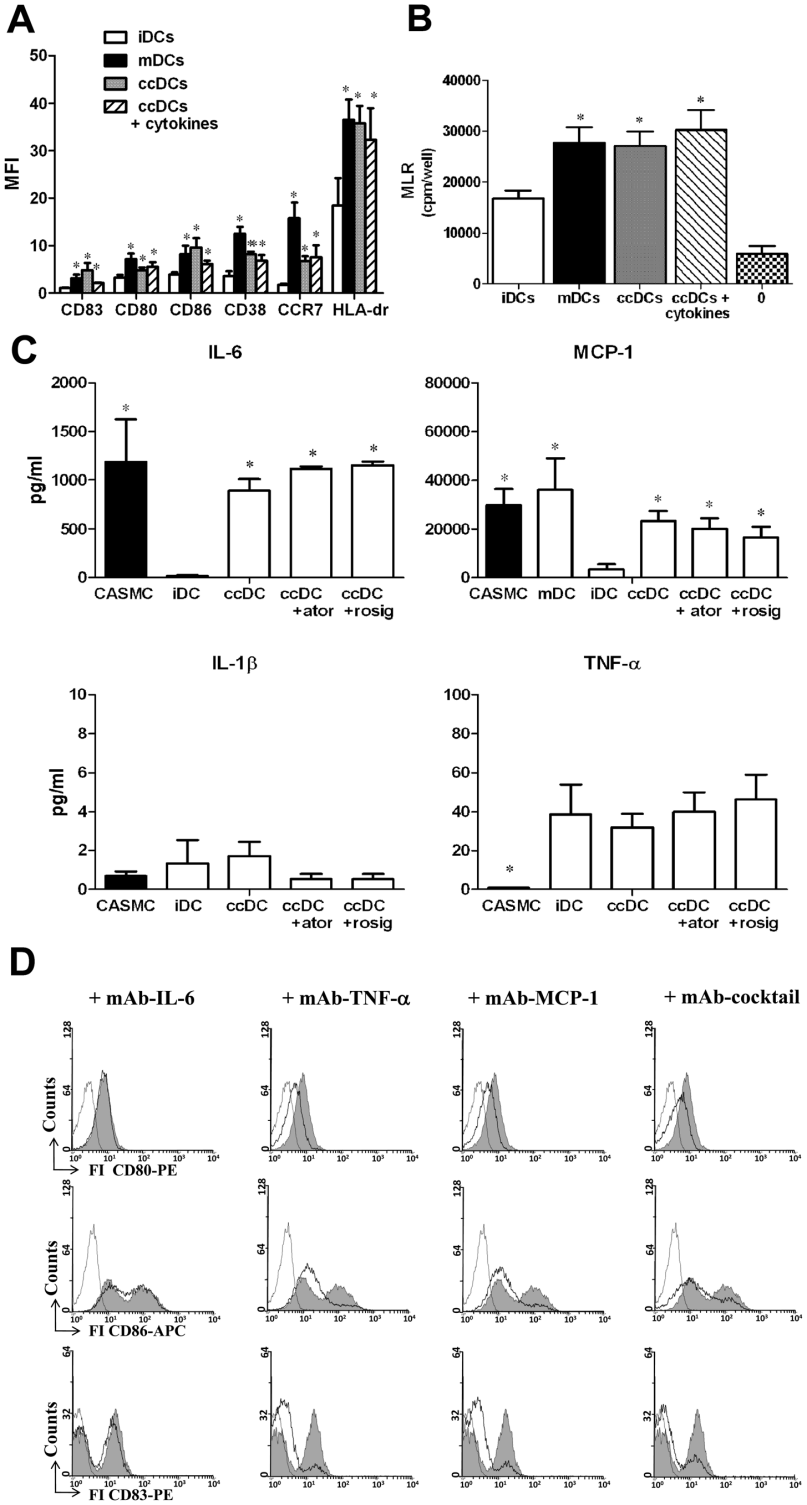
Immunophenotypical and functional maturation of DCs. **A**) Flow cytometry for DC markers in immature DCs (iDCs), DCs matured with a standard protocol (mDCs), DCs co-cultured with CASMCs (ccDCs) and DC co-cultured with CASMC pre-treated with 50 ng/mL TNFα and IFNγ (ccDCs + cytokines); all co-cultures were inside transwells. Mean ±SE of median fluorescence intensity (MFI), n = 12; *P<0.05 vs. iDC, ANOVA. **B**) Proliferation of lymphocyte alone (0) and in mixed reaction with iDCs, mDCs, ccDC or ccDCs + cytokines. Lymphocyte proliferation is expressed as mean ±SE of counts-per-minute/well (cpm/well; 4 experiments, each in triplicate); *P<0.05 vs. iDCs, ANOVA. **C**) IL-6, MCP-1, IL-1β and TNFα release from CASMC, iDCs, mDCs, ccDCs as assayed by Milliplex method. Atorvastatin (ator; 1 µmol/L) and rosiglitazone (rosig; 20 µmol/L) did not influence the release of cytokines in co-cultures. The release of cytokines was measured from 1×10^6^ cells DCs and 5×10^4^ CASMCs. Results are expressed as pg/mL. Mean ±SE of 4 experiments. *P<0.05 vs iDC, ANOVA. **D**) Flow cytometry analysis of DC maturation and of the effect of neutralizing antibodies against TNFα, MCP-1 and IL-6 or of a cocktail of all those antibodies. Representative histograms of ccDCs in the absence (filled histogram) and in the presence of antibodies (open histogram, black lines) are shown. The histogram for the isotype control is included (open histogram, faint line). The effect of non immune IgG is not shown.

After 36 h of co-culture with untreated CASMCs, iDCs significantly increased the expression of HLA-DR, the maturation markers CD83, CD38, CCR-7 and the co-stimulatory markers CD80 and CD86, indicating that they had undergone maturation (Fig.1 A and Fig. S1), similar to that achieved in DCs stimulated with cytokines (mDCs) which represented the positive control. Pre-treatment of CASMCs with IFNγand TNFα (50 ng/mL) for 24 h did not further increase the expression of co-stimulatory molecules and maturation markers by DCs. In order to evaluate the possible contribution of serum to DC maturation, new experiments were performed by setting co-cultures in RPMI medium in absence of FBS. After 36 h co-culture, flow cytometry analysis demonstrated increased expression of DC maturation markers similar to that obtained with co-culture in the presence of serum, despite an increase in cell death rate (9,53±1,3%. and 5,6±1,9 in the absence and in the presence of serum, respectively; Fig. S2).

In MLR, as expected, iDCs stimulated lymphocyte proliferation slightly (Fig. 1B). When they were co-cultured with CASMCs a significant increase occurred in the capacity to stimulate lymphocyte proliferation, comparable to that of mDCs; pre-treatment of CASMCs with TNFα or IFNγ did not further increase the lymphocyte stimulating activity of ccDCs.

Cytokines and chemokines possibly relevant to CASMC/DC interaction were measured in the supernatants of cell cultures. The results indicate that CASMCs produced quite high concentrations of IL-6 and high concentrations of MCP-1 in the absence of any stimulation (Fig. 1C). mDC also produced high concentrations of MCP-1. Co-culture of DCs and CASMC did not induce higher production of MCP-1 and IL-6 compared to CASMCs alone. Higher concentrations of TNFá were found in ccDC cultures compared to CASMCs alone, but these concentrations were similar to those secreted by immature DCs. IL-1â was produced at very low concentration by both iDCs and CASMCs, and the concentration did not increase significantly in co-cultures. Since TNFá, IL-6 and IL-1 were used to mature iDCs to mDCs, their concentrations in the medium of mDCs were not measured, in order to avoid possible misinterpretation. A positivity for IFNã was seen only in the positive control, but in none of the experimental cultures (data not shown). Given the anti-inflammatory properties of HMG-CoA reductase inhibitors [22] and of PPARγ agonists [23], their effect on cytokine release from co-cultures was measured: neither atorvastatin nor rosiglitazone influenced the release of cytokines in co-cultures (Fig. 1C).

In order to verify that cytokines released from the co-cultures were involved in DC maturation, blocking experiments were performed. The presence of monoclonal neutralizing antibodies against MCP-1 and TNFá partially inhibited iDC maturation, while the neutralizing IL-6 antibody had no significant effect (Fig. 1D). The observed inhibition was particularly evident for the maturation marker CD83, for which the mean fluorescence intensity (MFI) was 4.6±0.16, 4.5±0.17, 3.3±0.5, 3.4±0.51 and 3.4±0.16 for ccDCs, ccDCs plus anti-IL-6, ccDCs plus anti-TNFá, ccDCs plus anti-MCP-1 and ccDCs plus cocktail of antibodies, respectively (n = 3; p<0.05 by ANOVA). Non-immune IgG were ineffective (data not shown).

**Figure 2:**
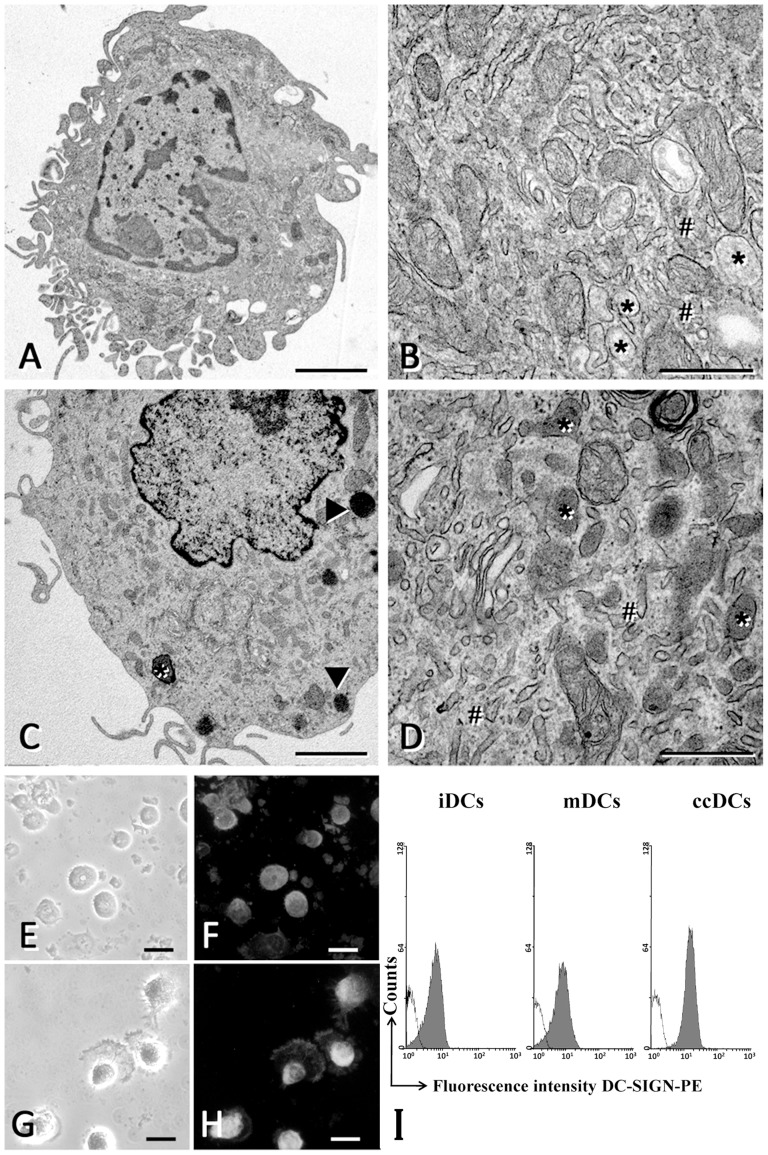
Characterization of co-cultured DCs. **A–D**) Electron microscopy of DCs matured in transwell culture with CASMC (A, B) or with cytokines (C, D). Asterisks indicate lysosomes; hashes indicate smooth endoplasmic reticulum; arrowheads indicate occasional lipid droplets. Bars  = 2 µm (A, C) or 0.5 µm (B, D). **E–H**) Phase contrast (E, G) and immunofluorescence microscopy (F, H) for DC-SIGN in immature (iDCs; E, F) and mature DCs (mDCs; G, H). The exposure time was the same for both F and H photomicrographs, to show that the labelling of iDCs was lighter than that of mDCs. Note the labelling on cell extensions, indicating membrane expression of the antigen. Bar  = 30 µm. **I**) Flow cytometry for DC-SIGN expression in iDCs, mDCs and ccDCs. One representative histograms out of 3 performed is shown. The isotype control is included (open histogram).

At electron microscopy, both mDCs and ccDCs were characterized by well developed smooth endoplasmic reticulum and Golgi apparatus, a few multivesicular bodies, and several lysosomes, most of which were small and with homogenous content (Fig. 2 A–D), as typical of well differentiated DCs. By immunocytochemistry it was shown that iDC expressed DC-SIGN faintly and mDC expressed this antigen intensely (Fig. 2 E–H), while neither iDCs nor mDCs expressed CD207/langerin (not shown); hence the cells had features of connective tissue DCs. Flow cytometry confirmed surface expression of DC-SIGN in iDCs, which increased during their maturation in either mDCs or ccDCs (Fig. 2 I).

### Soluble factors released by DCs stimulate CASMC to enhance migration

**Figure 3:**
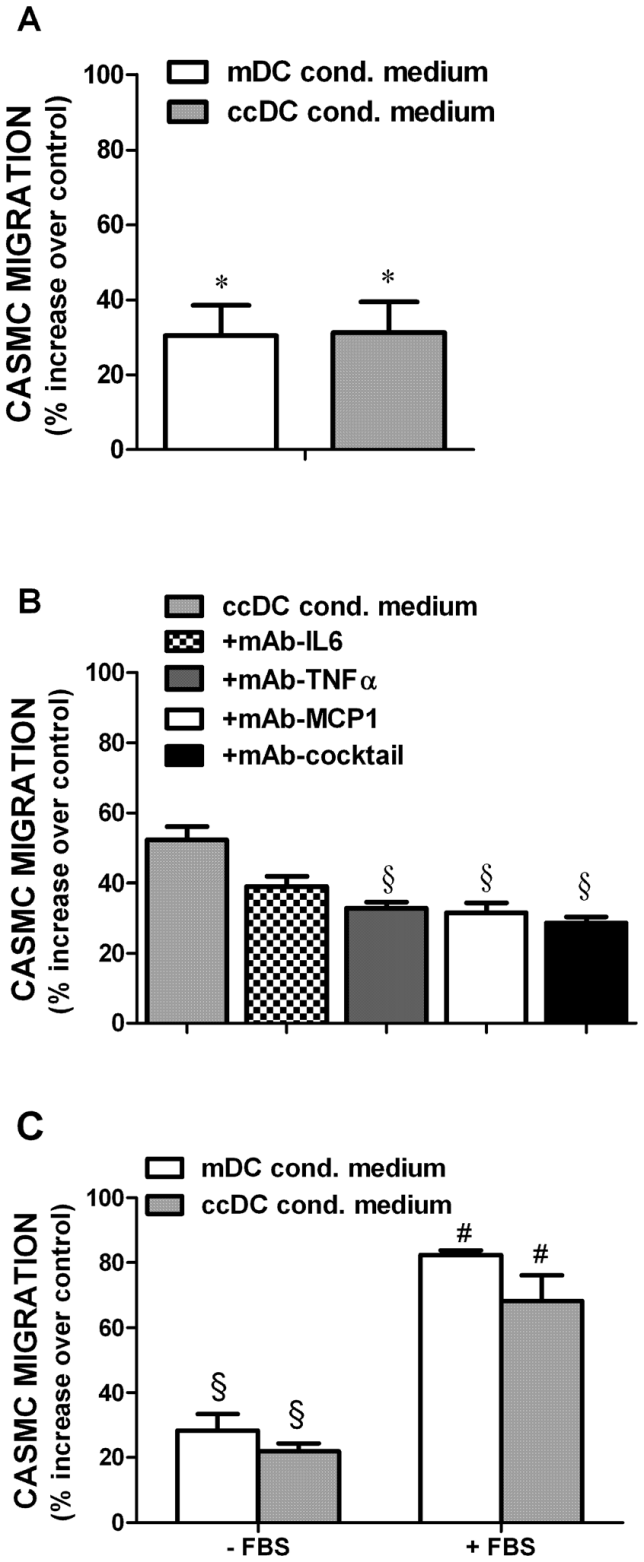
Influence of DCs on CASMC migration. **A**) Effect of DC conditioned media on CASMC migration. CASMCs were let to migrate in medium conditioned by mature DCs (mDCs) or by co-cultured DCs (ccDCs). Migration is expressed as percent increase over spontaneous migration of untreated CASMCs (control). Mean ±SE of 4 experiments, each in triplicate. *P<0.05 vs. control CASMCs, Student's *t* test. **B**) CASMC migration in response to medium conditioned by co-cultured DCs (ccDCs) in the absence or in the presence of neutralizing monoclonal antibodies against IL-6, (mAb-IL6, 1 µg/mL) TNFα (mAb-TNFα, 1 µg/mL), MCP-1 (mAb-MCP1, 2 µg/mL) or all antibodies together (mAb-cocktail). Migration is expressed as percent increase over untreated CASMCs (control). Mean ±SD of 3 experiments, each in triplicate. §P<0.001 vs. ccDCs, ANOVA. **C**) Effect of DC conditioned medium with or without FBS on CASMC migration. Migration is expressed as percent increase over spontaneous migration of untreated CASMCs (control). Mean ±SE (N = 16), each in triplicate. §P<0.001 vs unstimulated cells (control); #P<0.01 vs serum-free medium (-FBS) (ANOVA).

To test the hypothesis that DC/CASMC influence may be reciprocal, CASMCs were let to migrate in response to medium conditioned by mDCs or by ccDCs, in the absence of any DC. The migration of CASMCs was significantly enhanced by conditioned medium from either mDC or ccDC (Fig. 3A). Given the release of cytokines by ccDCs reported above, the effect of neutralizing monoclonal antibodies on CASMC migration was also assessed (Fig. 3B). The migration process of CASMCs induced by ccDC conditioned medium was significantly reduced by a cocktail containing antibodies against all the relevant cytokines (TNFá, MCP-1, IL-6). A significant effect on the inhibition of migration process was also detected using the neutralizing antibody against TNFα and MCP-1 alone, while the neutralizing IL-6 alone did not reach significance (Fig. 3B).

In order to evaluate the possible contribution of serum in the medium, CASMCs were let to migrate in response to medium conditioned by DCs (either ccDC or mDCs) grown in the absence of serum. CASMCs were still able to migrate in response to ccDCs- and mDCs-conditioned medium without FBS, although less intensely than when conditioned medium contained serum (Fig. 3C). Cell migration in response to medium conditioned by ccDCs was inversely correlated with the mortality of DCs in co-cultures without serum (Fig. S3).

### DCs adhere to CASMCs and binding is stimulated by an inflammatory microenvironment

**Figure 4:**
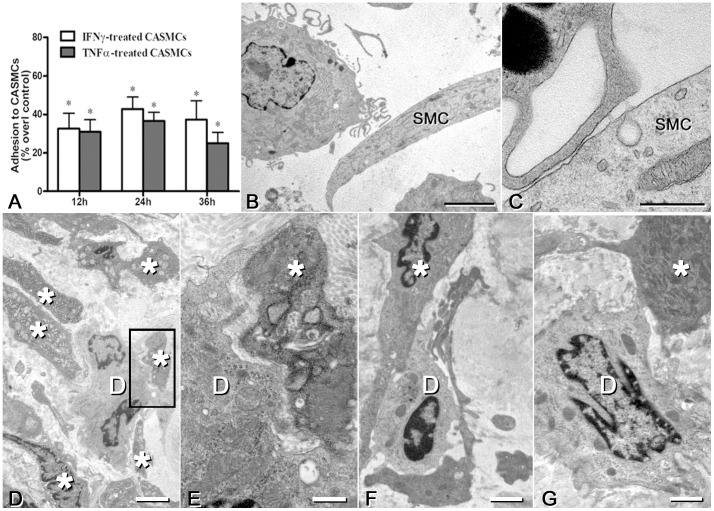
Adhesion of mDC to vascular smooth muscle cells. **A**) Human mature calcein-labeled DC adhesion to CASMC pre-treated for 12-36 h with 50 ng/mL IFNγ or with 50 ng/mL TNFα. After washing, a suspension of calcein-labeled DCs was added and let to adhere for 45 m. Mean ±SE of 6 experiments, each in triplicate. Results are expressed as percentage of mDC adhesion over that on untreated CASMCs (control). *P<0.05, ANOVA. **B, C**) Electron microscopy of mDCs adherent to citokyne-treated CASMCs; SMC: smooth muscle cells. Bars = 4 µm (B), or 0.5 µm (C). **D-G**) Electron microscopy of rat carotid repair tissue (neointima) at 7 (D, E), 14 (F) and 21 d (G) upon crush and incision injury; panel H is an enlargement of the boxed part of panel G. Dendritic cells (D) are seen in contact with smooth muscle cells with secretory phenotype (asterisks). Bar  = 2 µm (D), 500 nm (E), or 1 µm (F, G).

Untreated CASMCs bound human mDCs after 45 min incubation. Pre-treatment of CASMCs with TNFα or IFNγ (50 ng/mL) for 12, 24 and 36 h significantly increased mDCs adhesion to CASMCs (Fig. 4A). The maximal effect was obtained with 24 h pre-treatment. The DCs adhering to CASMCs contacted the latter cells through their dendrites, with small areas of close approach (≈20 nm) between membranes (Fig. 4B and C).

Adhesion between DCs and VSMCs with secretory phenotype was demonstrated also *in vivo*, in a model of carotid injury in the rat at all follow-up time points, *i.e.* 7, 14 and 21 days after injury (Fig. 4D-G). DCs were identified as endowed with cytoplasmic projections, variably rich in smooth vesicles and tubules, multivesicular bodies and intermediate filaments, and poor in lysosomes. Cells of smooth muscle lineage were identified as rich in microfilament bundles; they showed either a secretory or a contractile phenotype depending on whether microfilaments occupied less or more than half the cytoplasm.

**Figure 5:**
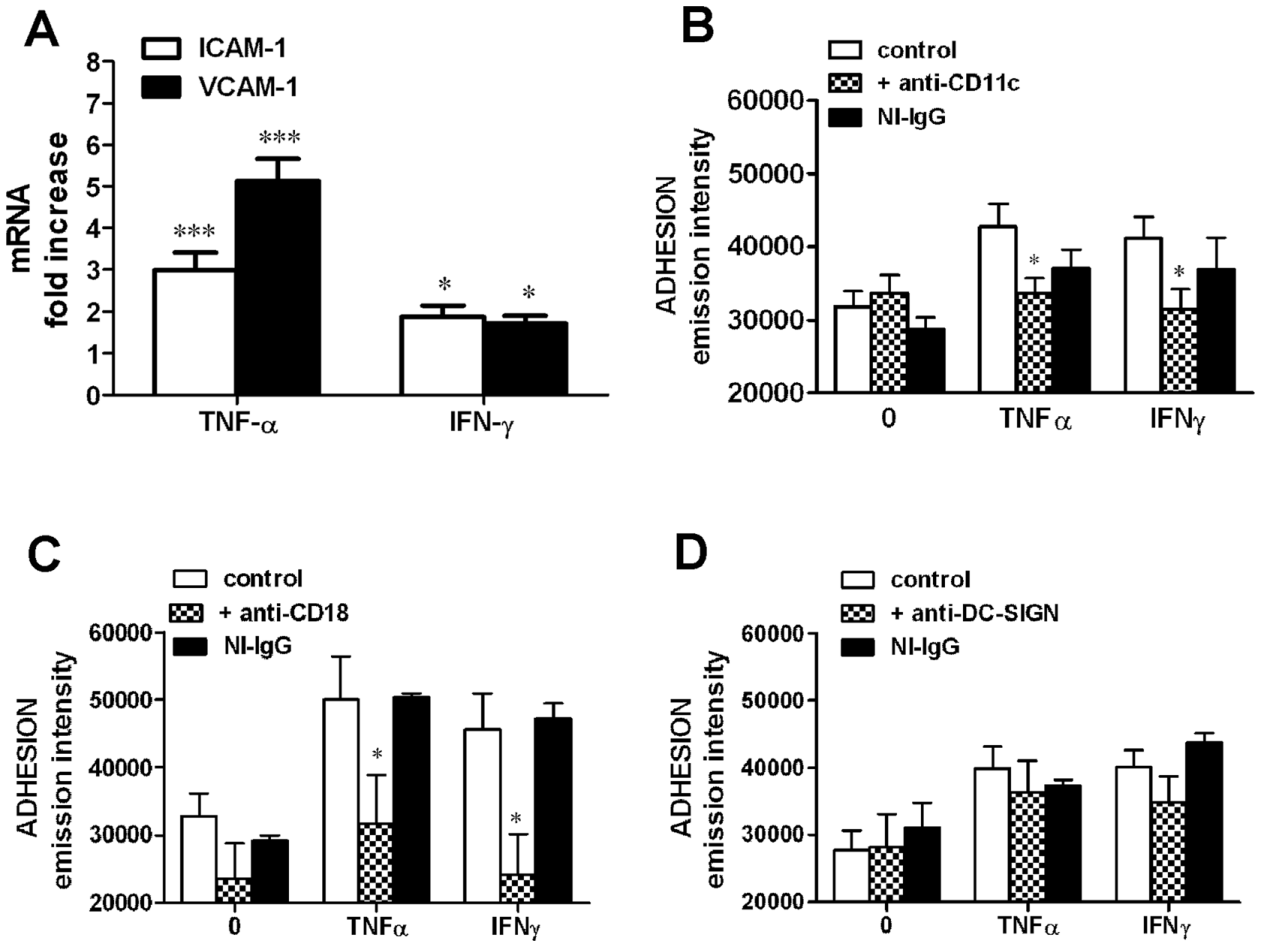
Adhesion molecules and counter-receptors involved in cytokine-induced mDC adhesion to CASMCs. **A**) real time RT-PCR of ICAM-1 or VCAM-1 mRNA expression by CASMCs stimulated with TNFα or INFγ (50 ng/mL each cytokine) for 24 h. Data are expressed as fold increase over unstimulated CASMCs. Mean ±SE of 3 experiments. *P<0.05, ***P<0.001 vs. untreated CASMCs, Student's *t* test. **B–D**) Effect of neutralizing antibodies on mDC adhesion to CASMCs. CASMCs were untreated (0) or pre-treated with: B) anti-CD11c (0.5 µg/well), C) anti-CD18 (0.5 µg/well), or D) anti-DC-SIGN (0.5 µg/well) neutralizing antibodies. They were pretreated with IFNγ or TNFα for 24 h and then washed before the assay. NI-IgG: non immune IgG. Mean ±SE of 5-7 experiments, each in triplicate. *P<0.05 vs. respective control, Student's *t* test.

In order to investigate the molecular mechanisms of adhesion between DCs and VSMCs in an inflammatory setting, we first studied the expression of ICAM-1 and VCAM-1 gene in CASMCs treated with TNFα or IFNγ by real-time PCR. Figure 5A shows that TNFα induced a significant increase of ICAM-1 and VCAM-1 mRNA expression by CASMCs, with maximal effects after 24 h stimulation. At the same experimental time point (24 h stimulation), IFNγ also significantly increased CASMC expression of ICAM-1 and VCAM-1 genes but to a lesser extent than that observed in response to TNFα. To confirm the involvement of VCAM-1 and ICAM-1 in CASMC/DC interaction we pre-treated mDC for 1 h with monoclonal neutralizing antibodies against CD11c and CD18, which are the counter-receptors of ICAM-1 and VCAM-1, and evaluated adhesion to CASMCs in comparison with that of DCs pre-treated with irrelevant IgG. Figs. 5B and 5C show that pre-treatment of DCs with anti CD11c or anti CD18 neutralizing antibodies significantly reduced their adhesion to CASMCs activated by either TNFα or IFNγ Given the expression of DC-SIGN by DCs and its proposed role in adhesion to endothelial cells, we also assessed the effect of a neutralizing antibody against DC-SIGN on mDC adhesion to CASMCs: this antibody proved ineffective at any concentration used (Fig. 5D).

### The adhesion between mDC and CASMC is responsive to drugs

**Figure 6:**
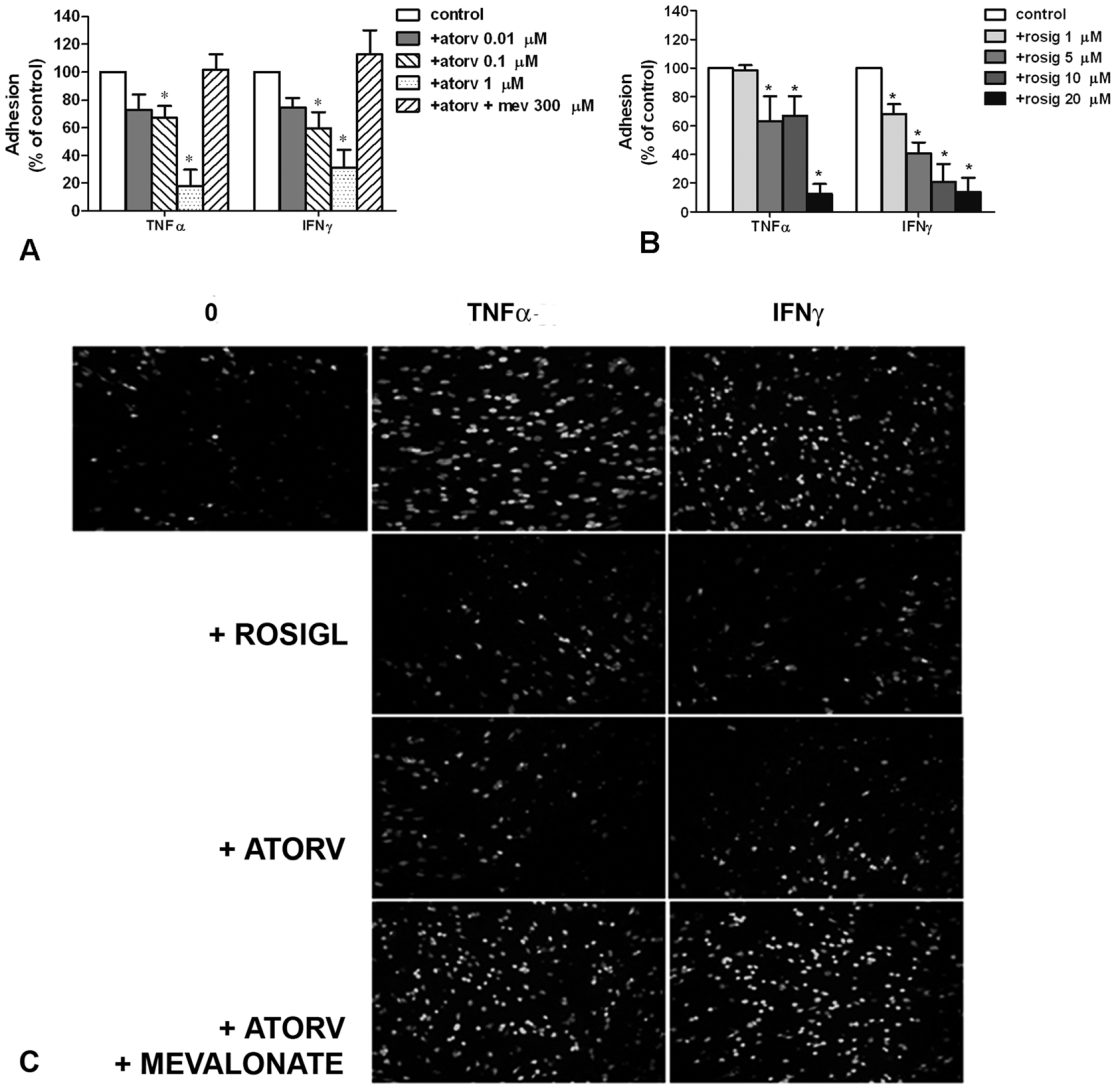
Effects of atorvastatin and rosiglitazone on mDC adhesion to cytokine-stimulated CASMCs. CASMCs were pre-treated with (**A**) atorvastatin (0.01–1 µmol/L) or (**B**) rosiglitazone (1–20 µmol/L) before stimulation with 50 ng/mL TNFα or IFNγ for 24 h followed by assay of DC adhesion as indicated for Figure 4. Mevalonate (300 µM), added to the maximal atorvastatin concentration, reverted statin effect (A). Adhesion is reported as percent of that induced by TNFα or IFNγ (control). Mean ±SE of 6–8 experiments, each in triplicate. *P<0.05 vs. control, ANOVA. (C) Representative experiment of DC to CASMC adhesion. Calcein-labelled mDC were incubated for 45 min with control CASMCs (0) or with CASMCs stimulated with TNFα or IFNγ (50 ng/mL either cytokine) and pre-treated, or not pre-treated, with 1 µmol/L atorvastatin (atorv) or 20 µmol/L rosiglitazone (rosigl). Mevalonate (300 µmol/L) was used to confirm atorvastatin selectivity.

The effect of atorvastatin and rosiglitazone was assessed on DC-CASMC adhesion by pre-treating CASMCs with atorvastatin (0.01–1 µM) or rosiglitazone (1–20 µM) for 1 h and then stimulating them with IFNγ or TNFα for 24 h before measuring adhesion to DCs. Adhesion was concentration-dependently inhibited by either atorvastatin (Fig. 6A) or rosiglitazone pre-treatment (Fig. 6B). The maximal effect was obtained with 1 µM atorvastatin and with 20 µM rosiglitazone, which nearly prevented the adhesion between the two cell types (Fig. 6). These highest concentrations used were devoid of any toxic effect on CASMCs, as revealed by trypan blue exclusion (data not shown). The dependence of atorvastatin effect on HMGCoA-reductase inhibition was confirmed by the addition of 300 µM mevalonate, which entirely prevented the inhibition of cell adhesion (Fig. 6A).

## Discussion

We have obtained evidence that interaction between human DCs and coronary smooth muscle cells (CASMCs) stimulates both the transition of human iDCs to a mature, efficiently immunostimulatory phenotype and the migration of CASMCs; reciprocal influence is exerted through soluble factors, which include MCP-1 and TNFα. The results upon co-colture in the absence of serum confirmed this reciprocal stimulation. We could also show that these cells adhere to each other with the intermediation of ICAM-1, VCAM-1 and their counter-receptor CD11c/CD18. An inflammatory setting, such as that represented by the addition of TNFα and IFNγ, stimulates DC adhesion to CASMCs. Factors released from mature DCs promote CASMC migration, while both the HMG-CoA reductase inhibitor atorvastatin and the PPARγ agonist rosiglitazone - known to have anti-inflammatory effects - counteract the cytokine-stimulated adhesion.

Adhesion between DCs and VSMCs could be replicated *in vivo* upon carotid injury in the rat; this experimental animal model, as previously demonstrated by Rinaldi et al. [23], is characterized by a significant inflammatory process. To the best of our knowledge, the present data are the first to demonstrate that DCs and CASMCs can stimulate each other by releasing soluble factors, hence they may cooperate in driving inflammation within the vascular wall, and that atorvastatin and rosiglitazone can interfere in such stimulation.

Few studies have investigated the involvement of DCs in vascular inflammation and remodelling. Feng et al.[24] demonstrated that ETS-1 transcription factor mediates inflammation and neointima formation in a model of carotid artery balloon injury, however they did not specifically addressed the behaviour and possible role of DCs even if it is known that ETS-1 is involved in DC adhesion [25].

Dendritic cells are specialized in antigen presentation and may also regulate inflammatory processes independent of immune mechanisms by stimulating the recruitment of neutrophils and NK cells and by secreting type I IFN [4], [26], [27]. Under physiological conditions the wall of human arterial branches down to at least 3–5 mm diameter hosts iDCs [6] - derived from circulating precursors - in the adventitia and among VSMCs where they are supposed to take care of surveillance against antigens as vascular-associated lymphoid tissue. DCs co-localize with neointimal VSMCs, besides T-cells, in human atherosclerotic lesions [6], [9] and in the rat carotid reacting to experimental injury (present data). Our data indicate that the co-localization is not a mere issue of tissue geometry, because they demonstrate that soluble factors released by CASMCs in the presence of iDC stimulate the latter towards a mature phenotype (mDCs), virtually identical to that achieved with a standard protocol and capable of stimulating allogeneic lymphocyte proliferation. Moreover, maturation of DCs was achieved independent of the presence of serum in the medium.

Among the soluble factors possibly responsible for DC/CASMC interaction, we paid attention to IL-1β, IL-6, IFNγ, TNFα and MCP-1, for several reasons. IL-1β can stimulate DC maturation [17], [28], is expressed in human and animal atherosclerotic lesions and has been demonstrated to stimulate VSMC migration [29]. IFNγ is found in human atherosclerotic lesions, stimulates VSMC adhesion molecule expression [30] and CASMC adhesiveness (present data). IL-6 is a biomarker of inflammation in cardiovascular diseases and stimulates maturation of iDCs [28], [30]. MCP-1 is a CC-chemokine secreted by vascular wall cells, which is involved in vascular wall remodelling [29], [31], [32] and in the maturation of DCs [33]. Our data show that IL-6 and MCP-1 are produced at quite high concentrations by CASMCs even alone, but at much lower concentration by iDCs. On the contrary, mDCs express huge amount of MCP-1 which in turn may mediate CASMC activation. TNFα too may play some role in CASMC stimulation, although its concentration in the medium of mDCs cannot be unequivocally interpreted since it is used to mature iDCs to mDCs. IL-1β was produced at very low concentration by both iDCs and CASMCs and the concentration did not increase significantly in co-cultures, so we would not propose a role for this cytokine in mediating intercellular influence. We confirmed an influence of CASMCs on iDC maturation with blocking experiments, which showed a decreased expression of CD83, CD80 and CD86 in co-cultured DCs (ccDCs) treated with neutralizing antibodies against MCP-1 and TNFα. Dendritic cells, either immature and mature, express chemokine receptors which are critical for their migration and maturation [33], [34], [35]. Our present data lend support to MCP-1 playing the latter role and to the interaction between DCs and VSMCs driving vascular inflammation.

No significant inhibition of DC maturation was observed in the presence of neutralizing antibody against IL-6, although it is known that IL-6 can stimulate DC maturation [28]. This result may be explained by the fact that in the co-culture medium there were other cytokines and chemokines together with IL6, in particular MCP-1 and TNFα (Figure 1C), which could be sufficient to cause DC maturation even when IL-6 effect was prevented.

Present data also demonstrate an enhanced DC adhesion to cytokine-pre-treated CASMCs, which appeared mediated by synthesis of ICAM-1 and VCAM-1 by CASMCs since the mRNAs for these molecules were increased upon culture with cytokines and, conversely, antibodies neutralizing their counter-receptors CD11c and CD18 significantly inhibited the event. Adhesion to cytokine-untreated CASMCs was not significantly affected by the antibodies, indicating that there are also other receptor-counter-receptors couples at work in basal conditions. It had already been demonstrated that VSMCs contribute to the retention of monocytes and neutrophils in the arterial wall [36], and previous studies had demonstrated an up-regulation of ICAM-1 and VCAM-1 mRNAs on intimal and media VSMCs of atherosclerotic vessels [37] and on isolated human smooth muscle cells [38], [39]. The present data point to TNFα and IFNγ as stimulating that up-regulation. We propose that VSMCs are implicated in the localization of DCs in the arterial wall (and not only of their monocyte precursors) and that ICAM-1 and VCAM-1 and their counter-receptors play roles in that localization. Afterwards, the interaction of VSMCs with DCs can concur to the final maturation and functional activation of the latter cells. Therefore we propose a role for that interaction in perpetuating and worsening the inflammatory condition leading to the progression and complication of atheroma and to vascular remodelling. It has been previously demonstrated that diabetes-like conditions stimulates VSMCs to bind human and mouse monocytic leukemia cells and to promote their differentiation to macrophages [40]. Also, DCs obtained from obese and diabetic (T2D) patients significantly increase their adhesion to CASMCs, compared to DCs isolated from either obese, non-diabetic patients or healthy subjects [21]. Since diabetes is a well known atherogenic disease involving - among others - inflammatory mechanisms, we propose that these mechanisms concur to activate DC-VSMC interactions and reciprocal activation also *in vivo*, as a step of the vicious cycle leading to atheroma progression and complication.

Dendritic cells present in an inflamed tissue also stimulate lymphocytes and other leukocytes to secrete pleiotropic molecules and the response of VSMC *in vivo* depends on all the stimuli they receive together. The present findings indicate that these stimuli include some ones directly given by DCs. These cells therefore are far more reaching than antigen presentation to lymphocytes, since they directly influence VSMC activation, which - at the best of our knowledge - is an original finding. Migration of CASMCs in response to medium conditioned by ccDCs or by mDCs in the absence of serum was less intensely stimulated than when the conditioned media were produced in the presence of serum. This may be due to less effective secretion of stimulating factors by DCs differentiated in the absence of serum, concomitant with the higher mortality of DCs in this condition.

A great number of studies performed also in patients [41] have shown that HMG-CoA-reductase inhibitors limit the progression of atherosclerosis and may even induce its regression and that glitazones are anti-inflammatory and anti-atherogenic agents besides anti-diabetic [42]. Several diverse mechanisms are at play, however their main consequence is a reduction in inflammation [22], [41], [42]. In the present paper we could demonstrate that pre-treatment of CASMCs with atorvastatin (0.01–1 µmol/L) or rosiglitazone (1–20 µmol/L) significantly impairs the enhancement of mDC adhesion to CASMCs in response to inflammatory cytokines. Given the pleiotropic and anti-inflammatory effect of statins [22], [43]–[45] and PPARγ agonists [23], we assessed whether these drugs influenced also the release of cytokines in co-cultures. This did not happen, so the effect on cell adhesion may be a direct one. Previous *in vitro* experiments had demonstrated that statins suppress a range of DC functions [46] and adhesion to VSMC should be added to the list. This may at least in part explain the finding that statin administration lowers DC number in atherosclerotic plaques [47]. Rosiglitazone hampers the formation of a neo-intima in the injured rat carotid [23] and another thiazolidinedione, ciglitazone, is able to impair oxLDL-induced DC maturation and function [48]; these effects of glitazones seem independent of those on glycaemia and may hint to a relevant role of PPARγ in atheroma formation and progression.

We are aware of the limitations of the *in vitro* model used here. However, no available model - as far as we know - allows to dissect the behaviour of neointimal smooth muscle cells from those of the intima, nor to have in culture high enough numbers of freshly isolated vascular smooth muscle cells for experiments in order to circumvent the possible effects of multiple passages *in vitro*. Therefore, we believe that the information gained from the present investigation may be helpful to better understand the cell interactions and the regulatory mechanisms during vascular injury response and should like to draw attention on what we feel as the two main original findings of this study: (1) co-localization of DCs and VSCMCs in atherosclerosis and upon injury not only can play a role in the retention of DCs in the tissue (as indicated by previous studies) but induce both the maturation of DCs and the migration of VSMCs; (2) an inflammatory microenvironment promotes adhesion between the two cell types. Therefore DC-VSMC interaction appears as a relevant link in the chain of events leading to complications of atherosclerosis and vascular surgery. Other points, as underscored above, are: (3) the addition of VSMCs to the cohort of cells directly responsive to DCs; (4) the identification of some putative molecular intermediates of the interaction, i.e. ICAM-1 and VCAM-1 among cell adhesion molecules and TNFα and MCP-1 among soluble factors; and (5) the finding that anti-inflammatory drugs endowed with an anti-atherogenic properties may play the latter role also through inhibition of DC-VSMC interaction.

## Supporting Information

Figure S1 **Flow cytometry analysis of co-cultured DCs.** Representative plots of cell surface antigens (filled histograms) expressed by human immature dendritic cells (iDCs), monocyte derived dendritic cells matured with cytokines (mDCs), and dendritic cells matured upon co-culture with human coronary artery smooth muscle cells in transwell inserts, without addition of standard cocktail of cytokines (ccDCs). The open histograms show the results with the isotype controls. In this experiment, dendritic cells ccDCs showed increased expression of CD80, CD83, CD86, CD38 and CCR-7 and HLA-DR similar to mDCs, which was not further increased by the addition of cytokines during co-culture (ccDCs+cytok).
(TIF)

Figure S2 **Flow cytometry analysis of co-cultured DCs in absence of serum.** Flow cytometry analysis of DC marker expression in DCs co-cultured for 36 h in absence (ccDCs – FBS) and in the presence (ccDCs + FBS) of serum. Mean fluorescence intensity (MFI) was calculated in gated cells excluding dead ones, i.e. excluding those positive for 7-AAD. Mean ±SE, n = 6.
(TIF)

Figure S3 **CASMC migration in response to medium conditioned by DCs.** DCs were co-cultured with CASMCs for 36 h in serum-free RPMI. DC death, as measured by flow cytometry, resulted on the average 9,53±1,3%. **A**) CASMC migration in response to medium conditioned by ccDCs with higher (>10%; N = 8) or lower (<10%; N = 8) death rate. Mean ±SE; *P<0.05 vs medium conditioned by ccDCs with death rate >10%, Student's *t* test. **B**) Correlation between CASMC migration and percentage of DC death rate. Spearman's rank correlation coefficient (ρ) was -0.4929, p<0.05 vs. ρ = 0.
(TIF)
