# Supplementary figures and images for: Stimulatory Interactions between Human Coronary Smooth Muscle Cells and Dendritic Cells
Source: PLoS One. 2014 Jun 16;9(6):e99652. doi: 10.1371/journal.pone.0099652 (PMC4059651; doi:10.1371/journal.pone.0099652)

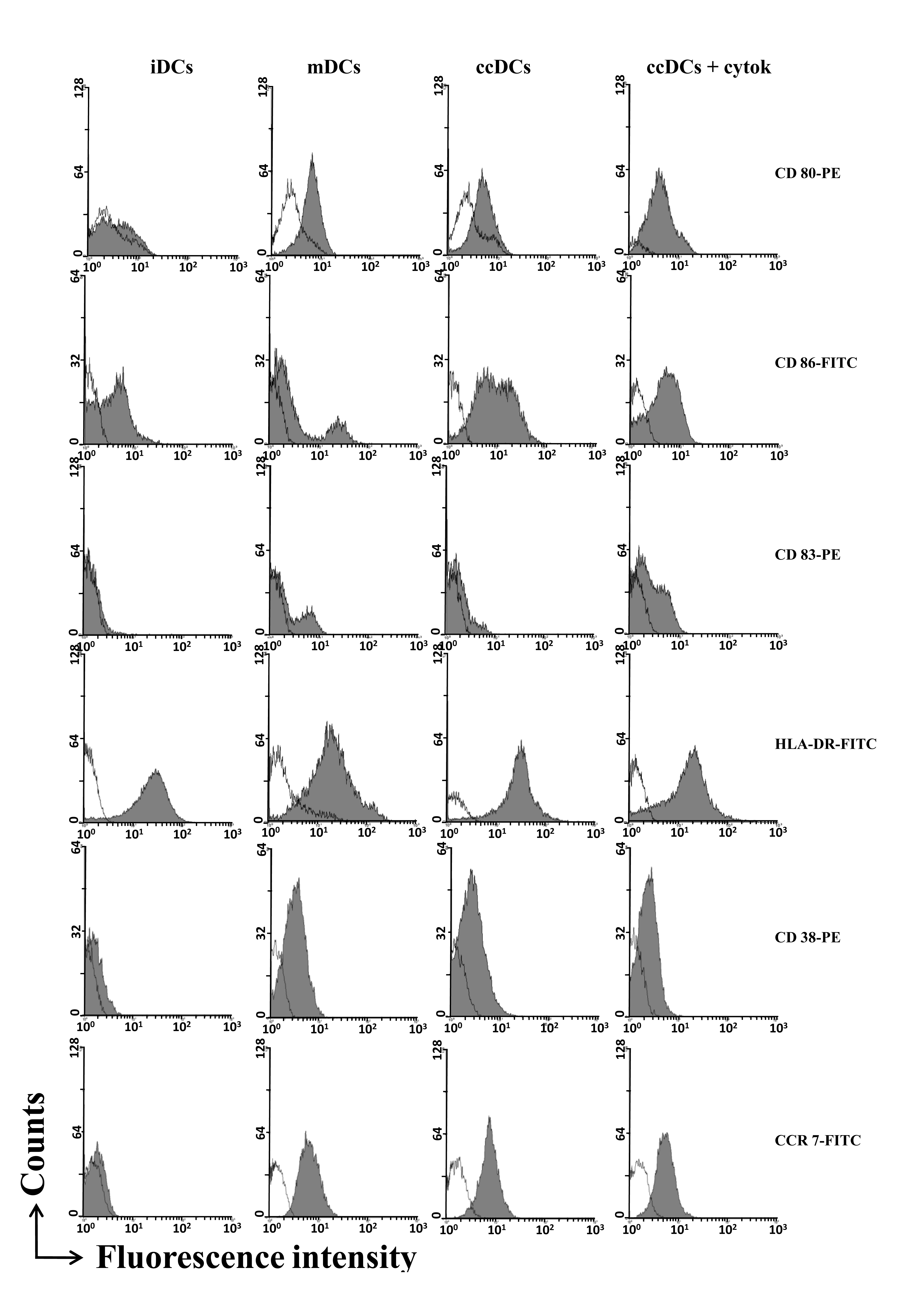

Supplement: Figure S1 — Flow cytometry analysis of co-cultured DCs. Representative plots of cell surface antigens (filled histograms) expressed by human immature dendritic cells (iDCs), monocyte derived dendritic cells matured with cytokines (mDCs), and dendritic cells matured upon co-culture with human coronary artery smooth muscle cells in transwell inserts, without addition of standard cocktail of cytokines (ccDCs). The open histograms show the results with the isotype controls. In this experiment, dendritic cells ccDCs showed increased expression of CD80, CD83, CD86, CD38 and CCR-7 and HLA-DR similar to mDCs, which was not further increased by the addition of cytokines during co-culture (ccDCs+cytok). (TIF) [file pone.0099652.s001.tif]
